# Editorial Topical Collection: “Biomedical Imaging and Data Analytics for Disease Diagnosis and Treatment”

**DOI:** 10.3390/bioengineering11070726

**Published:** 2024-07-18

**Authors:** Cosimo Ieracitano, Xuejun Zhang

**Affiliations:** 1 DICEAM Department, University Mediterranea of Reggio Calabria, via Zehender, Feo di Vito, 89122 Reggio Calabria, Italy; 2 School of Computer, Electronics and Information, Guangxi University, Nanning 530004, China; xjzhang@gxu.edu.cn

The integration of biomedical imaging techniques with advanced data analytics is at the forefront of a transformative era in healthcare [1]. This convergence has the potential to aid clinicians in disease diagnosis and treatment, offering significant insights and tools that are pivotal for improving patient outcomes [2]. Indeed, timely and accurate disease diagnosis is paramount to effective treatment planning and management.

Biomedical imaging modalities, such as magnetic resonance imaging (MRI), computed tomography (CT), positron emission tomography (PET), ultrasound, and optical imaging, are essential in modern medical practice [3]. These techniques provide invaluable visual information about anatomical structures, physiological functions, and pathological changes within the human body [4].

However, despite the advantages and capabilities of these imaging modalities, the sheer volume and complexity of the generated data pose significant challenges [4]. Clinicians and researchers often cope with the challenging task of extracting meaningful information from huge amounts of imaging data to make accurate diagnoses [5]. In this context, Artificial Intelligence (AI) has shown great promise in analyzing complex imaging data [6], identifying patterns [7], and making predictions [8,9] that can enhance diagnostic accuracy and treatment efficacy [10].

In this context, this Topical Collection includes thirteen papers focused on the latest advancements in biomedical imaging and data analytics for disease diagnosis and treatment. Each of the thirteen original contributions accepted for publication has undergone a rigorous review process, involving at least two expert reviewers and a minimum of two rounds of revisions. The studies, now published in the current Topical Collection, are briefly summarized as follows.

In Contribution 1, the authors present a review paper on existing label-free imaging technologies, particularly for middle-ear diseases, and explore the potential opportunities, barriers, and practical considerations for transitioning label-free technology to clinical applications.

In Contribution 2, the authors develop a speech enhancement model featuring a dual-path structure that identifies key speech characteristics in both the time and time–frequency domains. The experimental results showed that the proposed dual-path LSTM-based network outperforms conventional single-domain speech enhancement systems in terms of speech quality and intelligibility.

In Contribution 3, the authors exploit ResNet50, DenseNet201, InceptionV3, and InceptionResNetV2 networks for the differential diagnosis of Otitis Media with Effusion (OME) in pediatric tympanic membrane images. The trained networks demonstrated, on average, superior OME diagnostic accuracies compared to the performance of seven otolaryngologists, indicating their potential to assist in the clinical diagnosis of OME in pediatric patients.

In Contribution 4, the authors introduce the AMD long-term Information Viewer (AMD VIEWER), which provides comprehensive medical data display throughout an eye treatment course. The study results showed that regular visits resulted in better treatment outcomes, and AMD VIEWER proved significantly faster and error-free results compared to manual data input. With a Net Promoter Score of 70 from ophthalmologists, the tool is endorsed for improving data management and display for AMD patients.

In Contribution 5, the authors trained a 2D U-Ne to generate synthetic T1ρ maps from T2 maps for knee MRI, aiming to enhance datasets and enable rapid, reliable image reconstruction. The network, developed using 509 knee images from patients with ACL injuries, was evaluated on 343 clinical and 46 research knees, synthesizing high-fidelity T1ρ maps with a normalized mean square error of 2.4% and a Pearson’s correlation coefficient of 0.93. The study demonstrated minimal bias and quantification error below clinically significant thresholds, suggesting the potential for image synthesis to reduce acquisition time and standardize T1ρ as a biomarker for osteoarthritis.

In Contribution 6, the authors introduce a modified grey wolf optimizer with behavior considerations and dimensional learning (BCDL-GWO) for 3D registration using an affine transform, refined with the iterative closest point (ICP) method. The proposed algorithm produces high-quality 3D visualization images with small mean squared error, leading to the conclusion that the outcomes of BCDL-GWO with ICP are better than those from the statistical randomization-based particle swarm optimization (SR-PSO).

In Contribution 7, the authors propose a fully automated method to segment Bone Marrow Lesions (BMLs) and quantify BML volumes by means of a proposed deep learning-based system referred to as U-Net + InceptionResNetV2, reporting a Pearson’s correlation coefficient of 0.98, 2D DSC of 0.68, and 3D DSC of 0.60, using manual BML delineation as ground truth. Comparative evaluations showed that the proposed method outperformed other state-of-the-art methods on the same dataset.

In Contribution 8, the authors propose a novel EVO-MS model (a multiple stacking ensemble learning model optimized by the energy valley optimization (EVO) algorithm) to select the greatest number of informatics features for fibrosis quantification, reporting an accuracy of 0.864, a precision of 0.813, a sensitivity of 0.912, a specificity of 0.824, and an F1-score of 0.860, which demonstrates the effectiveness of the proposed EVO-MS model in staging the degree of liver fibrosis.

In Contribution 9, the authors examine the feasibility of using clinically acquired pelvic MRIs for visceral fat measurement by analyzing the correlation of visceral fat parameters at the umbilical and L5 vertebral body levels in Crohn’s disease patients. Significant associations were found for visceral fat index (VFI) and visceral fat ratio (VFR) between these levels, with good agreement between investigators and moderate to good agreement between analysis platforms. The findings suggest that the L5 level on pelvic MRIs can be a reliable reference for visceral fat quantification.

In Contribution 10, the authors introduce an innovative semi-supervised learning-based approach for the automatic classification of maxillofacial diseases, using a novel DNN model called WaveletFusion-ViT based on wavelet extraction and fusion modules to enhance performance. Preliminary results, evaluated through five-fold cross-validation, suggested that this approach may outperform traditional fully supervised models in classifying maxillofacial diseases with limited labeled samples, though further validation is needed.

In Contribution 11, the authors present a convexity-preserving level-set segmentation model tailored for precise segmentation of individual tumor organoids, addressing challenges such as intensity inhomogeneity and overlapping structures. The model integrates data-driven, curvature, and regularization terms to ensure accurate boundary convergence while maintaining convexity. the results of experiments involving pancreatic ductal adenocarcinoma organoid images demonstrated high segmentation accuracy with an average Dice value of 98.81% and efficient computation time (20.67 s), outperforming C-V and CPLSE models in both accuracy and speed.

In Contribution 12, the authors propose a deep learning pipeline using YOLO v5 models to detect and classify thyroid nodules from transverse and longitudinal ultrasound images, reporting improved sensitivity (84%) and specificity (63%) compared to traditional methods (sensitivity 76%; specificity 34%; *p*-value = 0.003).

In Contribution 13, the authors evaluate ChatGPT 3.5, CoPilot, and Gemini chatbots against the Patient’s Guide for delivering prostate cancer information based on expert-validated questions. ChatGPT 3.5 demonstrated superior performance in accuracy, timeliness, completeness, and understandability. CoPilot also performed well but slightly less so than ChatGPT 3.5. The study highlights the transformative impact of LLMs in healthcare education, emphasizing ongoing AI innovation and ethical considerations, especially in light of regulatory frameworks such as the EU AI Act.

In conclusion, this Topical Collection addresses various challenges in biomedical imaging and data analytics for disease diagnosis and treatment, emphasizing advanced computational methodologies applicable in healthcare settings. We extend our sincere appreciation to the Management Team of the *Bioengineering* journal for their unwavering support during the preparation of this collection. Special thanks are also due to all contributing authors and anonymous expert reviewers whose invaluable efforts ensured the selection of submissions of the highest quality.
